# Reference values for and interpretation of the Singapore Caregiver Quality of Life Scale: a quantile regression approach

**DOI:** 10.1186/s41687-020-00201-0

**Published:** 2020-05-06

**Authors:** Yin Bun Cheung, Shirlyn H. S. Neo, Grace M. Yang, Irene Teo, Geok Ling Lee, Debra L. M. Qu, Audrey R. X. Koh, Julian Thumboo, Hwee Lin Wee

**Affiliations:** 1 Program in Health Services & Systems Research, Duke-NUS Medical School, 20 College Road, Singapore, 169856 Singapore; 2 Centre for Quantitative Medicine, Duke-NUS Medical School, Level 6, Academia, 20 College Road, Singapore, 169856 Singapore; 3 Centre for Child Health Research, Tampere University, Arvo Ylpönkatu 34, 33520 Tampere, Finland; 4 Division of Supportive and Palliative Care, National Cancer Centre Singapore, 11 Hospital Drive, Singapore, 169610 Singapore; 5 Lien Centre for Palliative Care, Duke-NUS Medical School, 20 College Road, Singapore, 169856 Singapore; 6 Department of Social Work, National University of Singapore, 3 Arts Link, Singapore, 117570 Singapore; 7 Department of Rheumatology and Immunology, Singapore General Hospital, Outram road, Singapore, 169608 Singapore; 8 Saw Swee Hock School of Public Health, National University of Singapore (NUS), National University Health System (NUHS), 12 Science Drive 2, Singapore, 117549 Singapore; 9 Department of Pharmacy, Faculty of Science, National University of Singapore, 18 Science Drive 4, Singapore, 117559 Singapore

**Keywords:** Caregivers, Effect size, Reference values, Singapore Caregiver Quality of Life Scale

## Abstract

**Purpose:**

The Singapore Caregiver Quality of Life Scale (SCQOLS) was recently developed and validated in two languages - English and Chinese. The total and domain scores are scaled to range from 0 to 100. However, the scale is not at the interval-ratio level of measurement. To facilitate interpretation, we established the percentiles of the scale’s total and domain scores among family caregivers of patients with advanced cancers and demonstrate the effect size in terms of differences in relation to caregiver and patient characteristics.

**Methods:**

Data were drawn from a cross-sectional survey of family caregivers of patients with stage III or IV solid cancers in Singapore. Quantile regression was used to estimate the percentiles in relation to caregiver and patient characteristics.

**Results:**

Participants in adjacent categories of patient’s performance status and caregiver’s having other family members to share caregiving duties differed by 3 to 5 points in median quality of life total score and most domain scores (each Bonferroni-adjusted P, P[B], < 0.05). Ethnicity was associated with the Physical Well-being and Experience & Meaning domain scores (each P[B] < 0.05), with variable direction and magnitude. Education was associated with Mental Well-being and Financial Well-being (each P[B] < 0.05). Equations and examples for calculation of the percentiles are provided.

**Conclusion:**

Percentiles and effect size estimates are provided to facilitate interpretation of the SCQOLS.

## Introduction

Cancer is a disease that imposes major burden not only on patients but also on their family caregivers. There has been a shortage of caregiver quality of life (QOL) measurement scales [1, 2]. Our qualitative study of family caregivers of advanced cancer patients in Singapore, a multi-ethnic society in South-East Asia, has shown substantial differences between the concerns of the caregivers and the contents of existing QOL measurement scales developed in the West [3]. A study of the Chinese translation of the Caregiver Quality Of Life Index-Cancer conducted in China also concluded that there was “only partial support for the relevance and construct validity of the scale for Chinese caregivers” [4].

Recently we developed and validated the Singapore Caregiver Quality of Life Scale (SCQOLS) among family caregivers of patients with advanced cancers in Singapore [2]. The SCQOLS consists of five domains and 51 items in total, namely Physical Well-being (12 items), Mental Well-being (10 items), Experience & Meaning (12 items), Impact on Daily Life (13 items) and Financial Well-being (4 items). The items use a 5-point scale, from Not at All (0) to Very Much (4). Negatively worded items are recoded such that a higher score indicates a better QOL. The domain score is the mean of its item scores multiplied by 25 to scale to the 0–100 scale, after applying the “half-rule” to handle item non-response (if any). That is, item non-responses are replaced by the mean of the observed item scores in the same domain if there are responses to at least half of the domain items. A weighted sum of the domain scores gives a total score, using the number of items in the domains divided by the total number of items as the weight. The validity and reliability of the SCQOLS and its domains have been demonstrated [2]. The questionnaire is available in the English and Chinese language (https://www.duke-nus.edu.sg/lcpc/resources/scqols-request-form). We had also demonstrated, using an equivalence study approach and having conditioned on demographic and health covariates, that the two language versions gave mean scores within an equivalence margin of +/− 0.5 standard deviation [5].

The SCQOLS total and domain scores were scaled to range from 0 to 100. However, the scale is not at the interval-ratio level of measurement. To facilitate interpretation of what is a high or low QOL score, reference values are needed. Furthermore, tabulation of the differences in QOL scores in relation to caregiver and patient characteristics will provide effect size benchmarks for the interpretation of intervention effects and differences between persons.

Reference values, also called “norms” or “standards”, indicate the distribution of a measurement in a population. The concepts and methods for their development and clinical application are most elaborated in the assessment of fetal and child growth [6, 7]. Typically, a sample of “healthy” pregnancies/infants/children are recruited and the percentiles of an anthropometric measure in relation to personal characteristics (most commonly gestational age or age) are calculated. They are then used to “prescribe” what “normal” growth is, as opposed to using them to “describe” what the “observed distribution” is [6, 7]. The scale of the data collection operation is often large. One reason is that a large number of people has to be screened in order to identify a “healthy” sample. Another reason is that the conventional way to estimate characteristic-specific (e.g. age-specific) percentiles is to group the observations into categories (e.g. age intervals) and then calculate the percentiles for each category separately. Therefore, each category will need a sufficiently large sample size (≥ 200) [8]. This way of generating reference intervals ignores the fact that the observations from adjacent ordered categories are informative for estimating the percentiles in the flanked category. One response to this large sample size demand is to use broader categories, such that the multiplier for the sample size per category is smaller and therefore the total sample size required is more manageable [9]. This practice loses accuracy. For example, if 12-month age intervals are used, two children who are only 1 month apart may have different percentiles because they are at the upper and lower ends of adjacent age intervals, yet two other children who are 11 months apart may be shown to share the same percentiles because they are within the same age interval.

Modern research using regression or curve smoothing methods in the production of reference values substantially increase the level of precision or reduce the sample size requirements. Characteristics such as age are treated as continuous variables and pool all observations in the modelling [6, 7, 10]. Staffa et al. gave an introduction to the method in a medical context [11]. Cheung et al. applied this approach to the norming of cognitive function [9]. Using a regression-based approach for the estimation of reference intervals, a sample size of 70 has been considered the minimum acceptable sample size [10]. Furthermore, a sample size that exceeds 200 offers only minor incremental gain in precision [10]. Therefore, a sample size of at least 200 is a reasonable target. Furthermore, under this approach it is not mandatory to exclude “unhealthy” persons from the modelling. Percentiles can be obtained for both the healthy and unhealthy populations by including variables that differentiate them as predictors in the model that pools all observations. While the observations in the unhealthy population do not directly contribute information about the percentiles in the healthy population, they do contribute information about the effects of personal characteristics such as the age trend. It indirectly contributes to the accuracy in the percentile estimation for the healthy population. The potential of the regression approach does not seem to have been exploited in the construction of reference intervals for QOL measures.

We report our use of the quantile regression approach to the estimation of reference values for SCQOLS. Our primary aims are two-fold. First, we provide the regression equations to generate percentiles in relation to caregiver and patient characteristics. The equations can then be used to estimate the percentiles for a “mild” caregiver situation, similar to the “healthy” population in fetal and child growth, for prescription as the reference values to define what a poor or good QOL level is. Second, we provide the estimates of differences in median QOL scores between different levels of caregiver and patient characteristics. They serve as effect size benchmarks to facilitate the interpretation of the meaningfulness of a difference between persons or intervention groups. Our secondary aim is to demonstrate and discuss the use of a regression-based approach for constructing reference intervals in QOL research.

## Methods

### Study setting and design

Singapore is a multi-ethnic society. Chinese (74%), Malay (13%) and Indian (9%) are the major ethnic groups; English is the lingua franca whereas bilingualism is common [12]. The National Cancer Centre is the largest public provider of outpatient cancer care in the country, serving 65% of the cancer patients in the public sector [13]. The patients who need inpatient care are usually admitted to the Singapore General Hospital.

Details of the study design and procedures have been previously published [2]. Briefly, family caregivers of patients with advanced cancers who were receiving care from the National Cancer Centre or Singapore General Hospital were recruited. Participants must be 21 years of age or older, able to communicate in either English or Chinese (Mandarin), aware of the patient’s diagnosis, and the patients must have stage III or IV solid cancers. Consented caregivers were invited to self-administer the questionnaire in English or Chinese according to their own language preference. Some caregivers requested interviewer-administration. Informed consent was obtained before the survey. The Singapore Health Services Centralized Institutional Review Board approved this study.

The study comprised a baseline and a follow-up survey. The present manuscript only involves the baseline survey data, because the follow-up survey was only designed to estimate test-retest reliability. In addition to the SCQOLS and psychometric measures for assessment of the scale’s validity, the baseline survey also included items on demographics, caregiving and health background.

### Measurements

We consider four groups of predictor variables. (a) Caregiver demographics, including age, gender, ethnicity (Chinese, Malay, Indian and Others) and education (tertiary, secondary and primary or lower. (b) Survey procedures, including questionnaire language (English or Chinese) and mode of administration (self- or interviewer-administration). (c) A survey question that asked the caregivers whether s/he was “the only person”, “the primary person” or “one of the few persons” who carries out caregiving duties for the patient. They were coded as 0 to 2, respectively; a lower value is anticipated to predict a worse QOL. For brevity, we refer to this variable as “caregiver role”. (d) Patient characteristics, including performance status and cancer diagnosis. The performance status score ranged from 0 to 4, representing “without symptoms”, “with symptoms, fully ambulatory”, “with symptoms, in bed less than 50% during the day”, “with symptoms, in bed more than 50% during the day but not bedridden” and “bedridden”, respectively [14].

### Statistical analysis

Quantile regression was used to assess relationship between predictors and the 10th, 25th, 50th (median), 75th and 90th percentiles of each QOL score. By minimizing the sum of absolute deviation, as opposed to the least square regression that minimizes the sum of squared deviation, the quantile regression estimates the 50th percentile [6, 11]. This unweighted estimation is also called the median regression. By allocating appropriate weights to the deviation above or below the fitted curve, the corresponding percentiles are estimated [6, 11].

Following Bonferroni adjustment for multiplicity (five percentiles), a predictor was kept in the final model if its regression coefficient showed *P* < 0.01 in at least one of the five percentile equations in the initial multivariable model. Equivalently, this meant Bonferroni-adjusted P, or P[B], < 0.05. For ethnicity (four categories) and education (three categories), if at least one of the indicator variables showed *P* < 0.01 in the initial model, they were kept in the final model but categories that were not significantly different from each other (Wald test) would be combined. For levels of caregiver role and patient performance status (both ordinal variables), models treating them as categories or linear trends were both fitted. Since quantile regression is not based on maximum likelihood estimation, conventional methods for model selection are not available. For comparison of different models for caregiver role and patient performance status, we calculated the absolute deviations between the observed values and predicted median and compared the mean absolute deviation by paired-sample t-test. The simpler model (linear trend) was chosen if the difference was small and statistically non-significant. Since the analytic estimator of the standard error of quantile regression coefficients is known to be inaccurate, we used the bootstrap standard error with 100 replicates [6].

## Results

### Descriptive summary

Table 1 describes the study sample. A total of 612 caregivers were recruited. The mean age of the caregivers was 48 years; 61% were female; 85% were ethnic Chinese; 15% received primary education or below. About half of the participants chose to use the English questionnaire package; 90% self-administered the questionnaire. The percentage of caregivers who were the only, primary, and one of the few persons in the family who took care of the cancer patients were 21%, 35% and 44%, respectively. Patient’s performance status ranged from 0 (best) to 4 (worst). Colorectal (24%), lung (21%) and breast (12%) cancers were the major diagnoses; all other diagnoses had frequencies smaller than 12% and were combined as “Others”.

**Table 1:** Participant characteristics (*N* = 612) ^*^

Characteristics	Mean (SD) or N (%)^†^
Age (years)	48 (14)
Gender	
Female	373 (61.0%)
Male	239 (39.0%)
Ethnicity	
Chinese	521 (85.1%)
Malay	53 (8.7%)
Indian	19 (3.1%)
Others	19 (3.1%)
Education	
Tertiary	315 (51.5%)
Secondary	204 (33.3%)
Primary or below	93 (15.2%)
Questionnaire language	
English	304 (49.7%)
Chinese	308 (50.3%)
Mode of administration	
Self	551 (90.0%)
Interviewer	61 (10.0%)
Caregiver role	
0 Only person	126 (20.6%)
1 Primary person	217 (35.5%)
2 One of the few persons	269 (44.0%)
Patient’s performance status	
0 (Best)	71 (11.6%)
1	205 (33.5%)
2	81 (13.2%)
3	170 (27.8%)
4 (Worst)	85 (13.9%)
Patient’s diagnosis	
Colorectal	145 (23.7%)
Lung	129 (21.1%)
Breast	72 (11.8%)
Others	266 (43.5%)

^*^Mean and standard deviation (SD) for continuous variables; frequency (N) and percent for categorical variables

^†^Percentages may not sum to 100 due to rounding

### QOL total score

Table 2 shows the results of the initial quantile regression model. Age, gender, language version, mode of administration and cancer diagnosis had no association with any of the percentiles (each *P* > 0.01). Ethnic Malay participants were 13 points higher than ethnic Chinese participants in the 10th percentile (*P* < 0.01; significant after Bonferroni adjustment). Despite larger *P* values, Indian and Others also had higher 10th percentile than the ethnic Chinese. Higher QOL total scores in the non-Chinese ethnic groups were observed in most other percentiles. No statistically significant difference was found between Malay, Indian and Others in the five percentiles (each *P* > 0.10). Ethnicity was regrouped as Chinese and non-Chinese in subsequent analysis of the total scores. Primary/below education was associated with lower 90th percentile (*P* < 0.01). No statistically significant difference was found between tertiary and secondary education in the five percentiles (each *P* > 0.10). Education was dichotomized as secondary/above versus primary/below in subsequent analysis of the total scores.

**Table 2:** Percentiles of QOL total score, initial model

Predictor	10th	percentile	25th	percentile	50th	percentile	75th	percentile	90th	percentile
	Coef.	(95% CI)	Coef.	(95% CI)	Coef.	(95% CI)	Coef.	(95% CI)	Coef.	(95% CI)
Age										
(per 10 years)	1.3	(−0.9, 3.6)	1.3	(−0.5, 3.1)	1.1	(−0.1, 2.5)	1.7	(0.5, 2.8)	0.8	(−0.5, 2.1)
Gender										
Female	0		0		0		0		0	
Male	1.4	(−4.8, 7.7)	1.2	(−2.6, 4.9)	−0.4	(−3.5, 2.6)	−1.5	(− 4.0, 1.1)	− 2.1	(− 4.8, 0.7)
Ethnicity										
Chinese	0		0		0		0		0	
Malay	12.7^*^	(4.0, 21.4)	5.8	(−2.8, 14.5)	4.1	(−1.1, 9.3)	7.1	(0.3, 14.0)	4.5	(−1.1, 10.0)
Indian	13.6	(1.3, 25.9)	5.0	(−5.3, 15.3)	−0.9	(−8.9, 7.1)	3.2	(−6.1, 12.5)	0.1	(−6.5, 6.8)
Others	15.7	(3.0, 21.4)	14.7^*^	(4.5, 25.0)	8.4	(0.5, 16.4)	4.7	(−1.8, 11.3)	0.2	(−6.4, 6.9)
Education										
Tertiary	0		0		0		0		0	
Secondary	−4.0	(−9.7, 1.6)	−1.0	(−6.7, 4.8)	1.9	(−1.8, 5.5)	0.3	(−2.7, 3.3)	0.7	(− 2.5, 3.9)
Primary/below	−1.8	(−9.2, 5.7)	−3.9	(−10.0, 2.1)	− 3.0	(−7.7, 1.7)	−5.5	(− 10.2, −0.9)	−6.3^*^	(−9.9, − 2.7)
Language										
English	0		0		0		0		0	
Chinese	5.5	(0.3, 10.7)	3.2	(−7.8, 6.3)	1.2	(−2.2, 4.6)	1.3	(−2.0, 4.5)	−0.4	(−4.0, 3.3)
Mode of administration										
Self	0		0		0		0		0	
Interviewer	−1.2	(− 11.6, 9.2)	−0.8	(−7.8, 6.3)	−2.1	(− 7.7, 3.4)	−2.3	(−8.2, 3.7)	1.9	(−3.8, 7.6)
Caregiver role										
0	0		0		0		0		0	
1	4.5	(− 1.7, 10.7)	8.8^*^	(2.8, 14.8)	5.8	(0.9, 10.7)	4.3	(0.2, 8.3)	1.8	(− 2.7, 6.3)
2	12.5^*^	(6.3, 18.8)	15.1^*^	(10.2, 20.0)	9.8^*^	(4.3, 15.3)	7.3^*^	(3.4, 11.2)	3.6	(− 0.7, 7.9)
Performance status										
0	0		0		0		0		0	
1	−5.5	(− 16.7, 5.8)	−6.8^*^	(−11.9, − 1.8)	−4.2	(− 8.0, − 0.3)	−3.1	(− 6.3, 0.0)	− 2.0	(− 5.6, 1.6)
2	− 14.9	(− 27.7, − 2.1)	− 14.4^*^	(−21.3, − 7.4)	−8.0^*^	(−13.6, − 2.4)	−5.9	(− 11.0, − 0.7)	− 4.4	(−8.9, 0.2)
3	−16.3^*^	(−28.0, − 4.6)	−14.7^*^	(−20.1, − 9.2)	−9.7^*^	(−13.7, − 5.6)	−7.4^*^	(−11.1, − 3.8)	−5.7^*^	(−9.4, − 1.9)
4	−20.4^*^	(−32.3, − 8.5)	−22.2^*^	(−30.8, − 13.6)	−14.5^*^	(−20.6, − 8.5)	−8.9^*^	(−13.4, − 4.3)	−8.8^*^	(−13.8, − 3.7)
Diagnosis										
Colorectal	0		0		0		0		0	
Breast	5.0	(−5.2, 15.3)	0.3	(−6.6, 7.1)	1.1	(−4.8, 7.1)	0.3	(−4.3, 5.0)	3.6	(−2.1, 9.3)
Lung	8.6	(−0.1, 17.2)	2.2	(−3.3, 7.7)	0.8	(−3.4, 4.9)	−0.3	(−4.5, 3.9)	2.1	(−1.9, 6.2)
Others	6.3	(− 1.6, 14.3)	4.1	(−0.8, 9.1)	1.2	(−2.0, 4.4)	−1.0	(−3.7, 1.8)	−0.3	(−3.6, 3.1)
Intercept	36.4^*^	(15.3, 57.5)	47.2^*^	(5.3, 59.1)	61.3^*^	(51.4, 71.1)	65.9^*^	(55.4, 76.5)	76.5^*^	(66.3, 86.6)

^*^P < 0.01

For all five percentiles, caregiver roles and patient performance status showed a monotonic trend. The mean absolute deviation of the median regression model that kept caregiver role and patient performance status as categorical variables was 10.73. Treating caregiver role and patient performance as linear trend variables according to their numeric codes gave mean absolute deviation of 10.78. Paired sample t-test showed *P* = 0.318 for the difference. Hence the final model treated these two variables as quantitative variables with liner trends. The final model is presented in Table 3.

**Table 3:** Percentiles of QOL total score, final model

Predictor	10th	percentile	25th	percentile	50th	percentile	75th	percentile	90th	percentile
	Coef.	(95% CI)	Coef.	(95% CI)	Coef.	(95% CI)	Coef.	(95% CI)	Coef.	(95% CI)
Ethnicity										
Chinese	0		0		0		0		0	
Others	7.3	(0.6, 14.0)	6.1	(0.8, 11.5)	3.0	(0.2, 5.9)	3.3	(−1.5, 8.1)	3.9	(0.6, 7.1)
Education										
Secondary/above	0		0		0		0		0	
Primary/below	2.5	(−3.1, 8.0)	−1.1	(−6.7, 4.5)	− 3.2	(− 6.6, 0.2)	−4.7^*^	(−8.0, − 1.4)	−5.1	(− 9.1, − 1.0)
Caregiver role	5.7^*^	(2.2, 9.3)	6.4^*^	(4.0, 8.7)	3.1^*^	(1.0, 5.2)	1.8^*^	(0.7, 3.0)	0.6	(− 0.9, 2.2)
Performance status	−5.1^*^	(−7.7, − 2.6)	−4.7^*^	(−6.4, − 2.9)	−3.3^*^	(4.6, − 2.2)	−2.7^*^	(−3.3, − 2.1)	−2.2^*^	(−3.1, − 1.3)
Intercept	52.0^*^	(42.6, 61.5)	61.1^*^	(56.9, 65.2)	73.4^*^	(69.1, 77.6)	82.4^*^	(80.1, 84.6)	88.3^*^	(85.1, 91.4)

^*^P < 0.01

^†^Example: The 10th percentile of an ethnic Indian caregiver with tertiary education who is the only family member (caregiver role = 0) giving care to a patient who has symptoms but is ambulatory (performance status = 1) is 52.0 + 7.3 + 0 + (0 × 5.7) + (1 × [− 5.1]) = 54.2

### Domain scores

The final models for the five domains scores are presented in Tables 4, 5, 6, 7, 8. Similar to the prediction of the total score, caregiver role and patient performance status were predictors of all the domain scores except Experience & Meaning.

**Table 4:** Percentiles of QOL Physical Well-being score, final model

Predictor	10th	percentile	25th	percentile	50th	percentile	75th	percentile	90th	percentile
	Coef.	(95% CI)	Coef.	(95% CI)	Coef.	(95% CI)	Coef.	(95% CI)	Coef.	(95% CI)
Ethnicity										
Chinese	0		0		0		0		0	
Indian	−9.7	(−25.1, 5.7)	− 9.4	(−30.9, 12.1)	− 4.2	(− 18.9, 10.6)	0.0	(−8.4, 8.4)	1.0	(−6.3, 8.4)
Others	14.6^*^	(7.4, 21.7)	7.8	(1.7, 3.9)	6.3^*^	(2.2, 10.3)	4.2	(0.9, 7.4)	2.1	(− 0.4, 4.6)
Caregiver role	13.2^*^	(8.6, 17.8)	10.4^*^	(7.0, 13.8)	5.6^*^	(3.0, 8.1)	4.2^*^	(2.4, 5.9)	1.0	(− 0.6, 2.6)
Performance status	−4.9^*^	(−7.8, − 1.9)	−5.6^*^	(−7.9, − 3.6)	−5.6^*^	(−6.8, − 4.3)	−4.1^*^	(−5.3, − 3.1)	−2.1^*^	(−2.9, 1.3)
Intercept	41.7^*^	(30.4, 52.9)	62.5^*^	(55.1, 69.9)	81.9^*^	(76.6, 87.3)	91.7^*^	(87.5, 95.8)	97.9^*^	(94.9, 100.9)

^*^P < 0.01

^†^Example: The 10th percentile of an Indian caregiver who is the only family member (caregiver role = 0) giving care to a patient who has symptoms but is ambulatory (performance status = 1) is 41.7–9.7 + (0 × 13.2) + (1 × [− 4.9]) = 27.1

**Table 5:** Percentiles of QOL Mental Well-being score, final model

Predictor	10th	percentile	25th	percentile	50th	percentile	75th	percentile	90th	percentile
	Coef.	(95% CI)	Coef.	(95% CI)	Coef.	(95% CI)	Coef.	(95% CI)	Coef.	(95% CI)
Education										
Secondary/above	0		0		0		0		0	
Primary/below	−10.6	(−20.8, − 0.5)	− 10.0	(− 18.1, − 1.9)	− 10.8^*^	(−18.3, − 3.4)	− 6.7	(−13.9, 0.6)	0.6	(− 5.7, 6.9)
Caregiver role	6.9^*^	(2.2, 11.6)	1.6	(− 0.6, 5.6)	3.3	(0.2, 6.4)	2.5	(−0.4, 5.4)	2.5	(−0.8, 5.8)
Performance status	−4.4^*^	(−7.1, − 1.7)	−5.0^*^	(−7.0, − 3.0)	−3.3^*^	(−4.9, − 1.8)	−3.3^*^	(−5.2, − 1.5)	−3.1^*^	(−4.7, − 1.6)
Intercept	35.0^*^	(26.5, 43.5)	55.0^*^	(47.5, 62.5)	65.8^*^	(60.5, 71.2)	78.3^*^	(71.3, 85.4)	86.9^*^	(80.7, 93.0)

^*^P < 0.01

^†^Example: The 10th percentile of a caregiver who has tertiary education background and is the only family member (caregiver role = 0) giving care to a patient who has symptoms but is ambulatory (performance status = 1) is 35.0 + 0 + (0 × 6.9) + (1 × [− 4.4]) = 30.6

**Table 6:** Percentiles of QOL Experience & Meaning score, final model

Predictor	10th	percentile	25th	percentile	50th	percentile	75th	percentile	90th	percentile
	Coef.	(95% CI)	Coef.	(95% CI)	Coef.	(95% CI)	Coef.	(95% CI)	Coef.	(95% CI)
Ethnicity										
Chinese	0		0		0		0		0	
Others	10.4	(−0.5, 21.3)	12.5^*^	(4.1, 20.9)	15.5^*^	(8.0, 23.1)	12.5^*^	(8.2, 16.8)	4.2	(− 1.9, 10.2)
Intercept	37.5^*^	(33.7, 41.3)	47.9^*^	(44.9, 51.0)	63.6^*^	(6.4, 65.9)	75.0^*^	(72.7, 77.3)	87.5^*^	(84.0, 91.0)

^*^P < 0.01

^†^Example: The 10th percentile of a Chinese or Other caregiver is 37.5 or 37.5 + 10.4 = 47.9, respectively

**Table 7:** Percentiles of QOL Impact on Daily Life score, final model

Predictor	10th	percentile	25th	percentile	50th	percentile	75th	percentile	90th	percentile
	Coef.	(95% CI)	Coef.	(95% CI)	Coef.	(95% CI)	Coef.	(95% CI)	Coef.	(95% CI)
Caregiver role	8.8^*^	(4.6, 13.1)	7.1^*^	(2.9, 11.2)	3.7^*^	(1.0, 6.4)	2.3	(0.4, 4.3)	1.0	(−0.7, 2.1)
Performance status	−6.5^*^	(−9.6, − 3.5)	−7.1^*^	(−9.6, − 4.5)	−4.4^*^	(−5.7, − 3.2)	−2.7^*^	(−3.7, − 1.7)	−1.8^*^	(−2.6, − 1.0)
Intercept	46.2^*^	(36.6, 55.7)	67.9^*^	(60.2, 75.7)	84.6^*^	(80.3, 88.9)	92.7^*^	(89.8, 95.5)	98.0^*^	(95.7, 100.2)

^*^P < 0.01

^†^Example: The 10th percentile of a caregiver who is the only family member (caregiver role = 0) giving care to a patient who has symptoms but is ambulatory (performance status = 1) is 46.2 + (0 × 8.8) + (1 × [− 6.5]) = 39.7

**Table 8:** Percentiles of QOL Financial Well-being score, final model

Predictor	10th	percentile	25th	percentile	50th	percentile	75th	percentile	90th	percentile
	Coef.	(95% CI)	Coef.	(95% CI)	Coef.	(95% CI)	Coef.	(95% CI)	Coef.	(95% CI)
Age										
(per 10 years)	2.3	(−3.5, 8.0)	6.0	(0.6, 11.3)	5.5^*^	(2.9, 8.2)	2.2	(−0.1, 4.6)	0	(0, 0)
Education										
Tertiary	0		0		0		0		0	
Secondary	−12.2	(−27.4, 3.0)	− 16.4^*^	(− 28.8, − 4.0)	−6.8	(−14.5, 0.8)	− 1.7	(− 4.7, 1.3)	0	(0, 0)
Primary/below	−15.8	(−35.0, 3.4)	−27.1^*^	(−42.7, − 11.5)	−22.3^*^	(−33.0, − 11.5)	−14.7^*^	(−26.0, − 3.5)	0	(0, 0)
Caregiver role	9.0	(0.8, 17.3)	12.8*	(5.4, 20.2)	13.5^*^	(8.7, 18.2)	5.9^*^	(2.2, 9.6)	0	(0, 0)
Performance status	−5.3	(−11.6, 1.0)	−2.8	(−7.2, 1.4)	− 2.5	(− 4.7, − 0.2)	−0.6	(−1.9, 0.7)	0	(0, 0)
Intercept	20.6	(−14.2, 55.3)	21.1	(−10.4, 52.7)	42.3^*^	(25.6, 59.1)	78..2^*^	(63.2, 93.1)	100.0	(100, 100)

^*^P < 0.01

^†^Example: The 10th percentile of a 50-year old caregiver who has tertiary education and is the only family member (caregiver role = 0) giving care to a patient who has symptoms but is ambulatory (performance status = 1) is 20.6 + (5 × 2.3) + 0 + (0 × 9.0) + (1 × [− 5.3]) = 26.8

For Physical Well-being, Malay and Others had higher but Indians had lower 10th percentile than ethnic Chinese participants (Table 4). Among the non-Chinese groups, there was statistically significant difference in the 10th percentile (*P* < 0.005) and non-statistically significant differences in the other percentiles (each *P* > 0.10). Hence, we regrouped ethnicity to Chinese, Indian and Others (including Malay) for the percentile equations. Education was not associated with Physical Well-being.

For Mental Well-being, primary/below education was associated with a lower median (Table 5). There was no significant difference between tertiary and secondary education (*P* > 0.10) and hence they were combined.

For Experience & Meaning, none of the predictors were associated with the scores except ethnicity (Table 6). All three non-Chinese groups had higher scores than the Chinese. There was no significant difference among the three non-Chinese groups in any of the five percentiles (each *P* > 0.10) and hence they were combined.

Only caregiver role and patient performance status were related to Impact on Daily Life (Table 7).

Age was related only to the Financial Well-being score (Table 8). Caregivers with tertiary, secondary and primary/below education background showed a gradient in this domain. Furthermore, as previously reported [2], the Financial Well-being score showed a heavy ceiling effect (24.7%). Hence, the quantile regression equation for the 90th percentile had intercept 100 and the regression coefficients for the predictors were all zero.

### Percentiles in a “mild” state

We present the predicted QOL total score percentiles for caregivers who were ethnic Chinese (mode) with tertiary education (mode), who were the primary (but not the only) caregivers in the families (caregiver role = 1) and whose care-recipients had disease symptoms but were fully ambulatory (performance status = 1) as a “typical” caregiver in a “mild” state, analogous to the “healthy” sample in fetal and child growth references. According to Table 3, the predicted 10th percentile for this group of caregivers is

$$ {10}^{th} percentile=52.0+0+0+\left(1\times 5.7\right)+\left(1\times \left[-5.1\right]\right)=52.6. $$

**Fig. 1:**
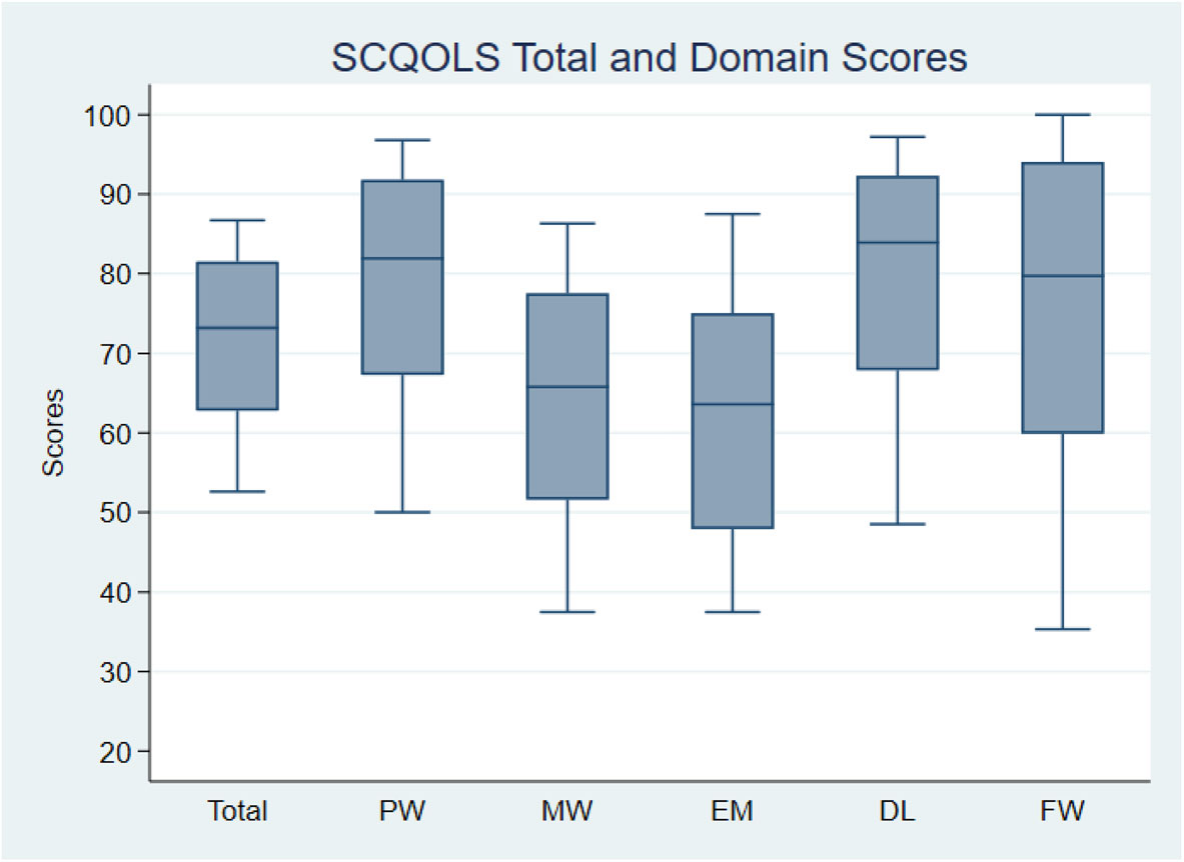
Plots of 10th, 25th, 50th, 75th and 90th percentiles of quality of life scores of caregivers in a “mild” state (patient performance status =1 and caregiver role =1) and, where applicable, in the most common demographic category (ethnic Chinese and tertiary education) and at mean age (48 years) in the survey. Calculation based on equations in Tables 3, 4, 5, 6, 7, 8. PW: Physical Well-being; MW: Mental Well-being; EM: Experience & Meaning; DL: Impact on Daily Life; FW: Financial Well-being

Following the same application of the equations in Table 3, the predicted 10th, 25th, 50th, 75th and 90th percentiles (standard error of prediction, not shown in Tables) of the “typical” caregivers in the “mild” state (*N* = 55) were 52.6 (2.5), 62.8 (1.3), 73.2 (1.0), 81.5 (0.7) and 86.7 (0.9), respectively (Fig. 1).

To compare the gain in precision by using quantile regression versus the conventional approach, we contrast the results for the caregivers above versus the percentiles estimated from the same group without using regression analysis. The percentiles (standard error) observed in this group of caregivers (*N* = 55) were 47.4 (5.6), 64.2 (2.3), 74.0 (2.5), 81.1 (2.7) and 89.6 (2.5), respectively. While the percentile estimates obtained from the two approaches were similar (except the 10th percentile), the standard errors were much smaller in the quantile regression approach.

Finally, based on the equations in Tables 3, 4, 5, 6, 7, 8, the percentiles of the QOL domain and total scores of caregivers in the “mild” state and, where applicable, in the most common demographic category (ethnic Chinese and tertiary education) and at mean age (48 years) are shown in Fig. 1.

## Discussion

With differences in socio-cultural context between the East and West and the sub-optimal validity of some caregiver QOL measurement scales for use in Asia [3, 4], the recent development of the SCQOLS is a valuable addition. Clinicians often need to classify test results in order to differentiate care recipients who require different types or level of services. In particular, it is important to identify care recipients who have an “abnormal” level of health indicators so as to provide timely interventions. Researchers may also have similar needs, such as to define inclusion/exclusion criteria for study enrolment and to define caseness endpoints. The percentiles we have presented can be used by clinicians and researchers of family caregivers to serve these purposes.

The use of regression analysis to estimate reference values tends to be more precise than the conventional way of separate calculation for each covariate interval or covariate combination. As we have seen in the illustration, the conventional way of estimating percentiles has substantially larger standard errors. The quantile regression is a precise and powerful tool for this purpose. Relative to other modern methods used in the construction of fetal and child growth references, quantile regression has the advantages of not involving a normal distribution assumption and ease of inclusion of multiple predictors.

The study sample’s demographic characteristics, such as education, age and gender, are similar to the caregiver profiles shown in other surveys of caregivers [15] and caregivers of cancer patients in Singapore [16]. As such, we believe the sample is representative of the target population.

We have previously provided evidence that the English and Chinese versions of the SCQOLS achieved equivalence in mean total and domain scores [5]. In the present analysis, we found no evidence of difference between the two language versions in any of the five percentiles in the SCQOLS total or domain scores. The availability of two language versions that have similar properties makes the SCQOLS suitable for use not only in Singapore but also in other countries where one or both of these languages are commonly used and in cross-country comparison.

Two surveys of the Singapore general population that used the Short Form 36 Health Survey showed that, having adjusted for covariates, ethnic Indian persons tended to score lower than other ethnic groups on the Physical Component Summary, but not the Mental Component Summary [17, 18]. While studies of general population do not directly shed lights on the caregiver population, they appear to be consistent with the present finding of ethnic Indian caregivers reporting lower level of Physical Well-being.

The Experience & Meaning domain captures strengths and sense making that caregivers may experience despite adversity [2]. We found that ethnic Chinese tended to score lower than the other ethnic groups in Singapore. A study of the caregivers of community-dwelling Singaporeans aged 75 years or above showed that ethnic Malay and Indian caregivers had higher level of Caregiver Esteem [19]. This may be a contributor to the observed difference in the Experience & Meaning domain.

A limitation of the present study is the relatively small number of non-Chinese caregivers. The estimates for the differences between ethnic groups should be considered tentative and will need further research to calibrate.

In the fetal and child growth context, samples are often limited to “healthy” population such that the percentiles derived are prescriptive instead of descriptive. For this reason, they are sometimes call growth “standards” instead of “reference values” [6]. Due to the variation in inclusion/exclusion criteria to define a healthy population, percentiles offered by a study may not fully satisfy the requirements of all users. The advantage of our approach to norming QOL is that the users can define a “mild” state flexibly. In our illustration, we focus on defining a “mild” state as (a) the care recipients being ambulatory despite having disease symptoms (performance status = 1) and (b) the caregiver being the primary but not the only person in the family to carry out caregiving duties (caregiver role = 1). The percentiles can be considered the QOL standards in a realistically favourable situation. While it is possible to select the best scenario (performance status = 0 and caregiver role = 2) to calculate the percentiles, it may not be realistic. Nevertheless, users of the SCQOLS and the quantile regression equations may do so if they consider it appropriate for their specific purposes.

We did not plan the study to define the minimal clinically important difference, the estimation of which usually requires longitudinal data with a sufficient amount of follow-up time to allow for changes in QOL to occur [20]. This is another limitation of our study. Nevertheless, the quantile regression results show us the differences in median QOL scores between important variables such as patient performance status and caregiver role. These two variables were predictive of most of the SCQOLS scores. Despite variations between domains, their adjacent categories mainly differed by about 3 to 4 points in the median scores. These differences can serve as benchmarks to help with the interpretation of whether an observed difference between persons or between intervention groups are practically meaningful.

## Conclusions

Percentiles and effect size benchmarks are available for the SCQOLS. They will facilitate the assessment of family caregivers in an Asian context.

## Data Availability

The de-identified dataset analysed is available from ScholarBank@NUS (10.25540/AXN8-5EKD).

## Abbreviations

QOL: Quality of Life
SCQOLS: Singapore Caregiver Quality of Life Scale
SD: Standard deviation
